# *Paenibacillus polymyxa* biofilm polysaccharides antagonise *Fusarium graminearum*

**DOI:** 10.1038/s41598-018-37718-w

**Published:** 2019-01-24

**Authors:** Salme Timmusk, Dana Copolovici, Lucian Copolovici, Tiiu Teder, Eviatar Nevo, Lawrence Behers

**Affiliations:** 1 Department of Forest Mycology and Plant Pathology, Swedish University of Agricultural Sciences, Uppsala, Sweden; 2 Faculty of Food Engineering, Arad University of Aurel Vlaicu, Arad, Romania; 3 Department of Plant Physiology, Estonian University of Life Sciences, Tartu, Estonia; 4 International Graduate Centre of Evolution, University of Haifa, Haifa, Israel; 5 National Academy of Sciences, Washington, USA; 6 Nova West Technologies & Communications, Tucson, AZ USA

## Abstract

Fusarium Head Blight (FHB) caused by *Fusarium graminearum* pathogens constitutes a major threat to agricultural production because it frequently reduces the yield and quality of the crop. The disease severity is predicted to increase in various regions owing to climate change. Integrated management where biocontrol plays an important role has been suggested in order to fight FHB. *P. polymyxa* A26 is known to be an effective antagonist against *F. graminearum*. Deeper understanding of the mode of action of *P. polymyxa* A26 is needed to develop strategies for its application under natural settings in order to effectively overcome the pathogenic effects. This study aims to re-evaluate a former study and reveal whether compounds other than non-ribosomal antibiotic lipopeptides could be responsible for the antagonistic effect, despite what is often reported. Wheat seedlings were grown to maturity and the spikes infected with the pathogen under greenhouse conditions. The development of FHB infection, quantified via the disease incidence severity and 100-kernel weight, was strongly correlated (r > 0.78, p < 0.01) with the content of the polysaccharide component D-glucuronic acid in the biofilm. Furthermore, while increased inoculum density from 10^6^ to 10^8^ cells/ml did not affect wild type performance, a significant increase was observed with the *P. polymyxa* mutant deficient in nonribosomal lipopeptide synthesis. Our results show that *P. polymyxa* A26 biofilm extracellular polysaccharides are capable of antagonizing *F. graminearum* and that the uronate content of the polysaccharides is of critical importance in the antagonism.

## Introduction

Fusarium head blight (FHB) is a devastating disease of cereals and is caused by a group of pathogens of which *F. graminearum* prevails in Nordic countries. The disease severity is predicted to increase in various regions owning to climate change^1^. FHB is of particular concern because many of the *Fusarium* species produce mycotoxins that contaminate infected grain and may pose a serious threat to human and domestic animal health. Grain that has been infected with the fungus may become incorporated into our staple diets. For these reasons the antifungal activity of biocontrol agents against FHB pathogens has been extensively studied and several mechanisms suggested^2–5^.

**Figure 1:**
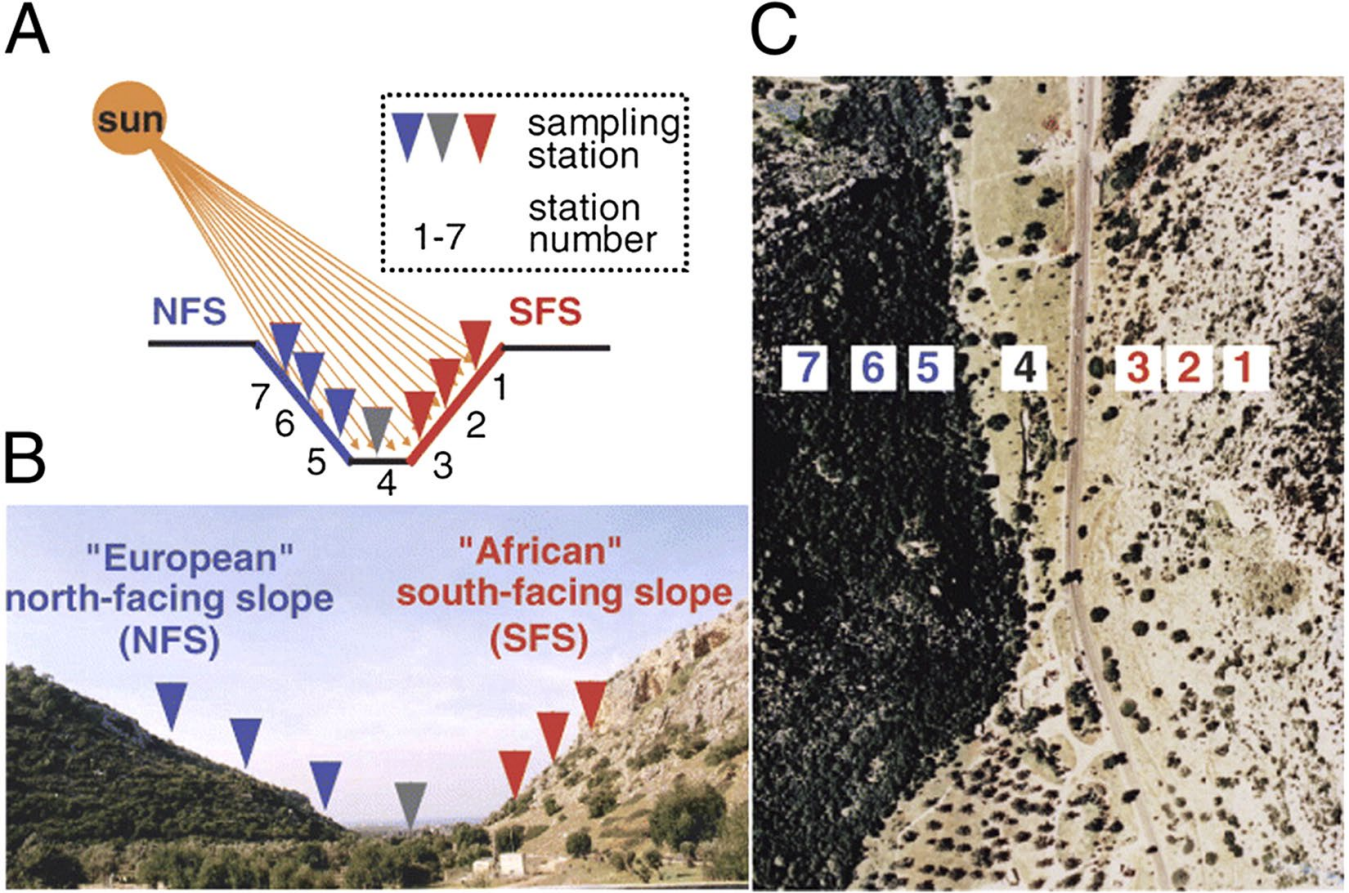
The Evolution Canyon (EC) model. (**A**) Schematic diagram. (**B**) Cross section view of EC at Lower Nahal Oren, Mount Carmel. (**C**) Air view of EC (source^10^: Nevo, 2012 Evolution Canyon,” a potential microscale monitor of global warming across life, PNAS 109; 8) (Photo by E. Nevo).

*Paenibacillus polymyxa* is known as an FHB biocontrol agent. It is generally recognised that surface-associated bacteria colonise as biofilms, which are microniches entirely different from their surroundings. This allows the bacteria to work as a functional unit, accomplishing tasks not possible for their planktonic state. Biofilms consist of cells and matrices where complex exopolysaccharides and proteins are major components, and they can provide an important bacterial survival strategy in natural systems^6–8^. *P. polymyxa* biocontrol ability is often linked to the large bacterial pool of bioactive compounds such as nonribosomal peptides/polyketides (NRPs/PKs)^4,9^. Despite enormous diversity the compounds have common regulatory systems, as they are produced nonribosomally and require activation by phosphopantetheinyl transferases (PPTase) of which Sfp-type PPTase is required for activation of peptidyl and acyl carrier domains. The broad range of activities of NRPs and PKs includes promotion of adaptation to unfavourable environments. The *P. polymyxa* A26 strain was isolated from the Evolution Canyon, South Facing Slope, Israel (EC SFS)^10,11^ (Fig. 1 and Table 1) and has co-evolved with wild progenitors of cereals over a long period of time, helping host plants to adapt and survive under diverse harsh conditions^11^. The genome analysis reveals regions that reflect these adaptations and the isolate is a superior plant growth promoting bacterium (PGPB) compared to isolates from more moderate environments^11^. In order to study the performance of the isolate we inactivated its Sfp-type PPTase gene and created a mutant that is incapable of producing enzymatically active 4′-phosphopantetheinyl transferase. This in turn results in a strain lacking enzymatically active NRPs and PKs (A26∆sfp), and the nonribosomal peptide and polyketides, often reported as active metabolites for the biocontrol^4,9^, are not produced.

**Table 1:** Strains and primers used in the study.

Name and abbreviation	Origin	Publications
*Paenibacillus polymyxa* A26 (A26)	**Wild barley rhizosphere**, Evolution Canyon, Haifa, Israel	Timmusk *et al*.^11^
*P. polymyxa* A26∆sfp (A26Sfp)	Wild barley **rhizosphere**, Evolution Canyon, Haifa, Israel	Timmusk, S. *et al*.^9^
*Fusarium graminearum A602/1998*	The National Veterinary Institute, Sweden	Abd El-Daim *et al*.^12^

Formerly we established a screening method for developing FHB biocontrol agents based on combining dual plate assays with a kernel assay^12^. Using the method, dual plate assays confirmed that NRPS/PKs products have critical importance for the antagonistic activity of A26. Studies with the more complex system employing a wheat grain assay showed, however, that in the case of one FHB-causing pathogen, *F. culmorum*, biofilm matrix formation may be the biocontrol agent of major importance in antagonising the pathogen. At the same time, only limited antagonism of the other pathogen, *F. graminearum*, was observed in the wheat grain assay^9,12^. This raised the question of why, in the case of *F. graminearum*, biofilm antagonism did not work and only the NRPS/PKs products were of critical importance.

The present study was conducted to re-evaluate the role of the *P. polymyxa* A26 extracellular matrix in antagonism to *F. graminearum*. The mutant (A26∆sfp), which lacks the ability for NRP and PK synthesis^9^, was studied under two inoculum densities, low (10^6^ cells/ml) and high (10^8^ cells/ml). We show that the mutant has comparable antagonistic ability to that of the wild type at the high inoculum density, and that the ability is correlated with the content of uronates in the biofilm polysaccharides. While many studies have been performed aiming to reveal the mechanism of antagonism of the economically important FHB pathogens, to the best of our knowledge this is the first report on the involvement of the uronate components of the biofilm polysaccharides.

## Material and Methods

### Experimental set-up

Three experimental series were performed:

1. The wheat grain assay was performed at two inoculum densities in order to re-evaluate the role of the A26 extracellular matrix in antagonizing *F. graminearum*. A26∆sfp was applied at low and high inoculum densities (10^6^ and 10^8^ cells/ml respectively). The results clearly showed the superior effect using the higher inoculum density.
2. Developing biocontrol agents requires combining screening strategies. Hence, greenhouse experiments on wheat plants grown to maturity were performed to test the effect in more natural settings. Wheat head assays evaluating disease severity (DS), disease incidence (DI) and 100-kernel weight (100-kw) were performed. We were further interested in the involvement of exopolysaccharides (EPS), which are the largest group of biofilm components, and along with wheat head assays their biofilm EPS assessment was performed.
3. In order to confirm the role of EPS in disease suppression, A26∆sfp and A26 EPS assays were performed on wheat kernels along with assays of supplied levan and alginate. Commercial samples of levan and sodium alginate were used as reference substrates owing to their contrasting uronate contents (Table S1).

### Microbial growth and culture conditions

*P. polymyxa* A26 was isolated from the South Facing Slope at the natural laboratory called the Evolution Canyon, Israel (Table 1). The Sfp-type 4-phosphopantetheinyl transferase deletion mutant strain was previously generated as described by us earlier^9^. Stock cultures were stored at −80 °C and were streaked for purity on half strength tryptic soy agar (1/2 TSA, pH 6.2). 10 ml of half strength tryptic soy broth (1/2 TSB, pH 6.2) was inoculated with single cells and grown at 30 ± 2 °C for 12 h. 100 µl of the preinoculum with 10^8^ cells per ml was used for 100 ml flask culture inoculations. Bacterial strains were grown in 1/2 TSB, pH 6.2, 180 rpm at 30 ± 2 °C, for 72 h. Cultures were centrifuged and pellets resuspended in sterile water as described earlier^12^. Finally, the cultures were adjusted to 10^6^ and 10^8^ cells/ml.

The fungal pathogen *F. graminearum* strain A602/1998 was obtained from the National Veterinary Institute, Uppsala, Sweden and was previously characterised for virulence^12^. The pathogen was grown on potato dextrose agar (PDA) plates for seven days at 22 °C. Macroconidia for inoculation were obtained by flooding the surface of colonized agar with sterile or phosphate buffered saline (PBS). The resulting inoculum density of the suspension was 10^5^/ml.

### Plant treatment

Winter wheat (Stava) seeds were surface sterilised by a 60 s wash in 99% ethanol, followed by a 6 min wash in 3% sodium hypochlorite solution, a wash in 99% ethanol, and repeated rinsing in sterile water. The seedlings were grown in greenhouse soil in 30 cm diameter pots. After a month of growth, the plants were vernalized for two months. Throughout the growing season the plants were watered daily with a standard nutritional solution. The experiment was performed in four replicates, each consisting of 20 plants. Wheat heads were sprayed with 100 µl of bacterial solutions at either low or high inoculum density (10^6^ and 10^8^ cells per ml respectively) at the beginning of the flowering stage (BBCH 61). The control plant heads were treated with 100 µl of sterile water. One week after the antagonist treatment (end of flowering stage) 30 µl of macroconidia suspension was used to inoculate a single central floret on each wheat head (four replicates each containing twenty plants). Controls were heads treated only with water or with pathogen suspension. Inoculated spikes were misted with water and covered with plastic bags for 10 days. Immediately before the pathogen inoculation, A26 and A26∆sfp biofilms from twenty wheat heads from each treatment were carefully flushed into 10 ml sterile water in 50 ml Falcon tubes for the biofilm studies. As controls, untreated wheat heads were flushed with sterile water. Counting of bacterial colony forming units was performed on ½ TSB plates. The biofilm bacteria were re-isolated by selective plating and confirmed by PCR as described by us earlier^13^. Wheat heads were collected 21 days after inoculation at the fully ripe stage (BBCH 89) and scored for disease incidence and severity on a scale from 0 to 100%. Disease severity was evaluated based on visual assessment of heads exhibiting FHB disease symptoms. *F. graminearum* presence was randomly confirmed using PCR^12^ and fluorometric assay. After the FHB evaluation the heads were allowed to dry and 100-kernel weights were determined.

### Wheat head biofilm assessment, and polysaccharide and glucuronate content evaluation

Wheat heads were flushed with 10 ml deionised sterile water immediately before the pathogen treatment in order to collect the biofilms developed after the A26 and A26∆sfp inoculations. The biofilm bacteria were re-isolated by selective plating, confirmed by PCR as described^9,13^, quantified, the supernatant polysaccharides isolated and D-glucuronate (D-GA) contents recorded. The biofilm samples in water were used for polysaccharide isolation as described earlier^14^. The pellet was lyophilised, weighed and stored at −4 °C until uronic acid content was assessed. The assay was based on hydrolysis of glucoside bonds that bind the polysaccharides. This step digests the polysaccharides into their component monosaccharides. Analysis of uronic acid content was performed as described earlier by Mojica *et al*.^15^ with small modifications. Briefly, the biofilm pellets were weighed and dissolved in 200 µl of deionised water. Potassium sulfamic acid (4 M, pH 1.6) was added to the biofilm solution and mixed by vortexing. Then sodium tetraborate solution in concentrated sulfuric acid (0.0125 M) was added. The solutions were incubated for 5 min in a 100 °C water bath, cooled on ice for 3 min and centrifuged at 2,000 rpm for 10 min after which 20 µl hydroxyphenol solution (0.15% v/v) was added to the supernatant. The solution was then mixed gently and the absorbance read at 520 nm. Each data point represents the average of twenty replicate measurements.

### *P. polymyxa* A26∆sfp cell and polysaccharide assay on wheat grains

The experiments were performed as reported earlier^12^. Briefly, conical flasks containing 20 g wheat grains were inoculated with 15 ml A26∆sfp at 10^6^ or 10^8^ cells/ml. For the polysaccharide assay, the kernels were treated with 15 ml of A26∆sfp and A 26 polysaccharide solution with the titres 10 µg/ml. Cells were grown and polysaccharides isolated as indicated below. Controls were treated with sterile water. In order to study the effect of uronates, two reference polymers contrasting in uronate content were applied: alginate (Sigma) and levan (Sigma). The polysaccharide powders were dissolved in deionized sterile water to the final concentration 15 µg/ml. 20 g wheat grains were mixed with 15 ml of the polysaccharide solution.

Flasks were incubated at room temperature for 8 h, and then inoculated with 1 cm^2^ agar plugs from 2 week old cultures of *F. graminearum*.

### EPS extraction

EPS extraction was performed as described earlier with small modifications^14^. Briefly, bacterial cultures were diluted 1:5 with distilled water and centrifuged for 30 min at 17,600 g at 20 °C to separate cells. Then, EPS were precipitated by slowly pouring the supernatant into two volumes of isopropanol while stirring at 200 rpm. The filtered polysaccharide was suspended in a digestion solution consisting of 0.1 M MgCl_2_, 0.1 mg/ml DNase, and 0.1 mg/ml RNase solution, and incubated for 4 h at 37 °C. Samples were extracted twice with phenol-chloroform, lyophilised using a Virtis SP Scientific 2.0 freeze dryer, taken to the initial volume and dialysed against distilled water.

### DNA extraction and quantification

The grain samples were freeze-dried and ground into a fine powder using a Precellys 24 homogenizer (Bertin Technologies, France). Samples were lysed by incubating 100 mg powder for 15 min in 350 μl of glucose buffer as described by us earlier^16^. DNA was extracted using a hexadecyl-trimethyl-ammonium bromide-based method^17^. The bacterial strains were confirmed by PCR as described by us earlier^13^. Fungal growth was assessed visually and randomly verified using PCR and sequencing after 10 days of growth^12^. For *F. graminearum* PCR ITS1F and ITS4 primers^18^ (5′-CTT GGT CAT TTA GAG GAA GTAA-3′ and 5′-TCC TCC GCT TAT TGA TAT GC-3′) were used. Initial denaturation at 95 °C was followed by 35 amplification cycles: denaturation at 95 °C for 30 sec, annealing at 58 °C for 30 sec, and extension at 72 °C for 30 sec, followed by an extension step of 72 °C for 7 min. The reaction mix contained 200 µm dNTP, 2.75 mM MgCl_2_, 0.025 U/µl polymerase (DreamTaq Green, Thermo Scientific, Waltham, MA, USA) and 0.2 µM of each primer. For the pathogen quantification the dsDNA of the PCR mix was purified using a Nucleo Spin kit (Macherey-Nagel, PA, USA) and quantified using the Invitrogen™ Qubit™ 4 Fluorometer according to the manufacturer’s instructions.

### Qualitative determination of biofilm composition by UHPLC-MS+

The chromatographic analyses have been performed using a liquid chromatograph (Nexera X2, Shimadzu, Tokyo, Japan) equipped with a diode array detector (M30A, Shimadzu, Tokyo, Japan) and a mass spectrometer (Model 8040, Shimadzu, Tokyo, Japan). The separation of compounds was performed on Nucleodur 100-5-NH2-RP column (4.6 mm i.d. × 250 mm column length, 5 µm particle size, Macherey-Nagel GmbH, Duren, Germany). The column temperature was maintained at 35 °C and the flow rate at 1 ml min^−1^. The solvents used for the chromatographic elution consisted of ultra-pure water with 0.1% TFA (A) and acetonitrile (B). The chromatographic elution program used was an isocratic one, with 25% A and 75% B, for 25 minutes. The injected volume of sample and standards was 10 µl. The DAD detector spectra were recorded between 200 and 600 nm. The mass spectrometer was equipped with an electrospray ionization (ESI) source operated in positive ion mode, and quantification was carried out in the multiple reaction monitoring (MRM) mode. The mass range was between m/z 15 and 1990. The ion spray temperature was maintained at 250 °C. The drying gas flow rate was 10 L/min.

### Evaluation of A26∆sfp and A26 EPS and sodium alginate water holding capacity (WHC)

Bacterial cultures were grown and harvested, and polysaccharides isolated, as described above. 600 mg of A26∆sfp and A26 EPS, sodium alginate (Sigma) and levan (Sigma) were mixed with 100 g sand and determined at different wetting cycles for 24 h. The sand and biopolymer mixture was then allowed to drain for 30 min. and the weight after saturation was recorded. Following the wet weight estimation the mixture was dried in an oven, cooled in a desiccator and weighed again. Each experiment was carried out in triplicate. WHC= gain in weight at saturation point/dry weight of soil × 100.

### Data confirmation and validation

To ensure reproducibility, greenhouse experiments were performed in four replicates each containing twenty plants. Twenty biological replicates of each D-glucuronate detection experiment were performed. For the wheat grain assay three biological replicates were performed. Replicated data were studied for normal distribution and analyzed by Unscrambler X15.1 and MiniTab17. One way analysis of variance (ANOVA) and a post-hoc LSD test was used to identify treatments that were significantly different from controls at p ≤ 0.05. Linear regressions (Unscrambler X15.1) were used to determine the relationships between antagonistic parameters and D-glucuronic acid content.

## Results

### *P. polymyxa* A26∆sfp antagonism against *F. graminearum* by kernel assay

**Figure 2:**
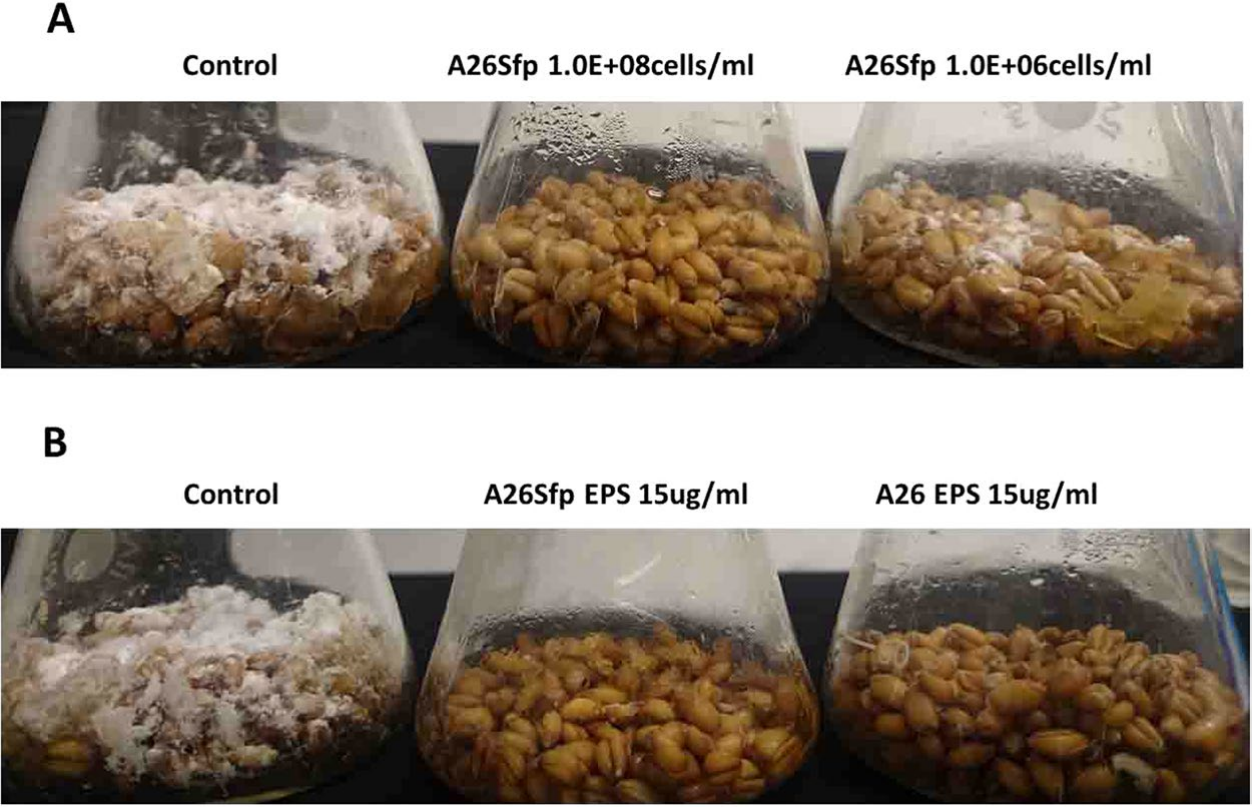
*Fusarium graminearum* antagonism in kernel assay. (**A**) Antagonistic activity of *Paenibacillus polymyxa* A26∆sfp at two inoculum densities, 10^6^ and 10^8^ cells/ml; (**B**) Antagonistic activity of *P. polymyxa* A26∆sfp and A26 extracellular polysaccharides (EPS) 15 µg/ml after 10 days incubation. See Material and Methods.

The experiment was performed following our former results on A26∆sfp *F. graminearum* wheat grain assay where grains inoculated with A26∆sfp showed limited pathogen antagonism^12^. Two initial A26∆sfp inoculum densities, 10^6^ and 10^8^ cells/ml, were used in the assay. Visual inspection of wheat grains over the experimental period revealed *F. graminearum* mycelial overgrowth in the control treatments. While limited mycelial growth was observed with A26∆sfp at 10^6^ initial inoculum density, no fungal mycelial growth was observed at 10^8^ A26Sfp inoculum density (Fig. 1A and Table S1). The wheat grain assay was carefully performed under axenic conditions in order to avoid contaminants from outside. *F. graminearum* growth randomly confirmed by PCR assays followed by Qubit^TM^ Fluorometer quantification revealed up to 0.3 ng/µl quantities of the pathogen DNA versus more than 10 ng/µl of the *F. graminearum* control treatment in the case of high initial inoculum (10^8^). Similarly to the assay performed by us earlier^12^, *F. graminearum* was detected around 3 ng/µl at low inoculum density (10^6^). The results show that the A26 mutant, deficient in nonribosomal compound synthesis, is still capable of efficiently antagonising the FHB-causing pathogen *F. graminearum* in the kernel assay when provided at 10^8^ inoculum density (Fig. 2A and Table S1).

### Antagonistic activity of *P. polymyxa* A26∆sfp and *P. polymyxa* A26 in the greenhouse assay

**Figure 3:**
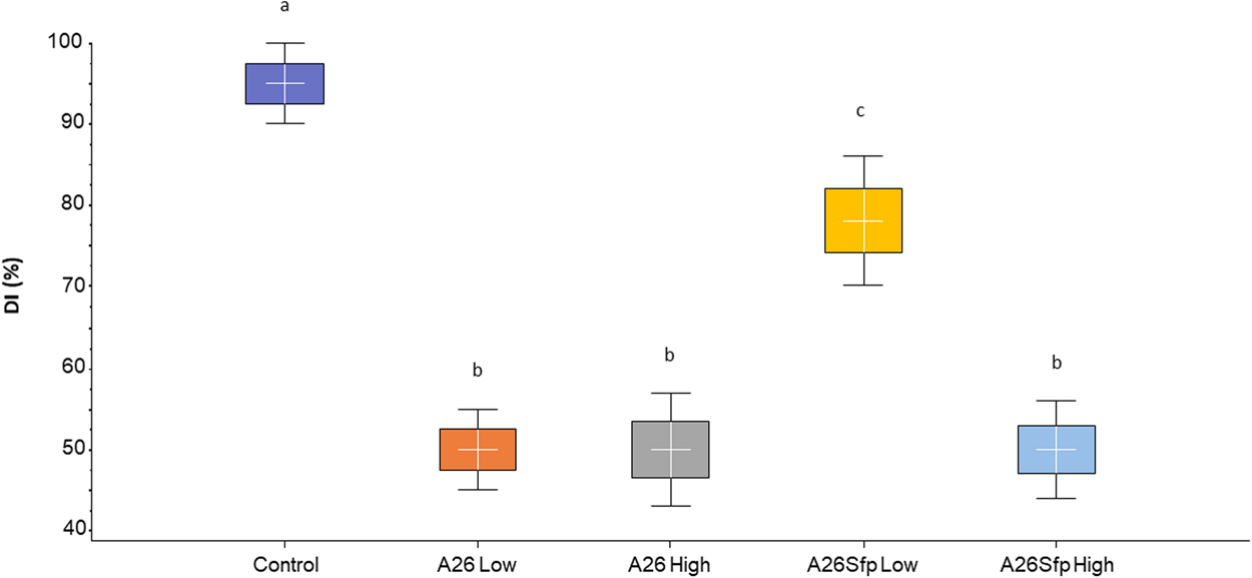
Fusarium Head Blight disease incidence (DI) in greenhouse assay. Boxplot of DI scores of wheat plants after *Paenibacillus polymyxa* A26 and *P. polymyxa* A26∆sfp treatments at two inoculum densities, 10^6^ (Low) and 10^8^ (High) cells/ml. ANOVA univariate analysis was performed and post-hoc LSD tests used to identify treatments significantly different from pathogen control (p < 0.05). Different letters indicate statistically significant differences. Error bars represent standard deviations.

**Figure 4:**
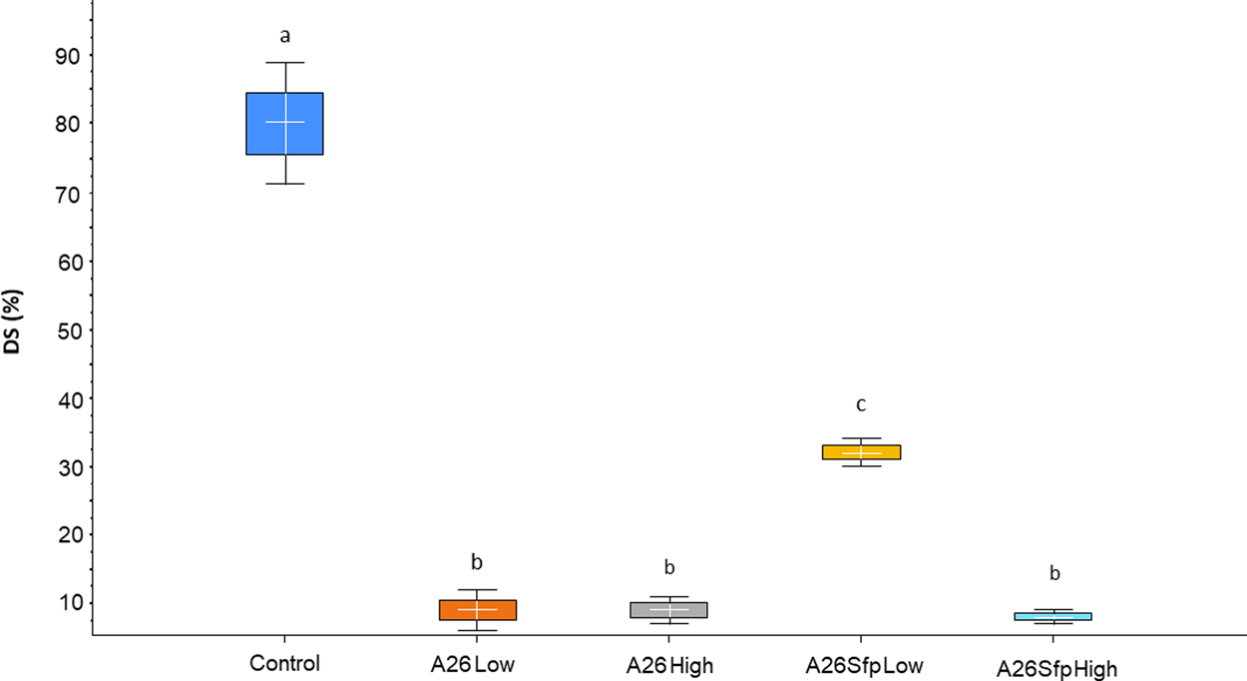
Fusarium Head Blight disease severity (DS) in greenhouse assay. Boxplot of DS scores after *Paenibacillus polymyxa* A26 and *P. polymyxa* A26∆sfp treatments at two inoculum densities, 10^6^ (Low) and 10^8^ (High) cells/ml. ANOVA univariate analysis was performed and post hoc LSD test used to identify treatments significantly different from pathogen control (p < 0.05). Different letters indicate statistically significant differences. Error bars represent standard deviations.

**Figure 5:**
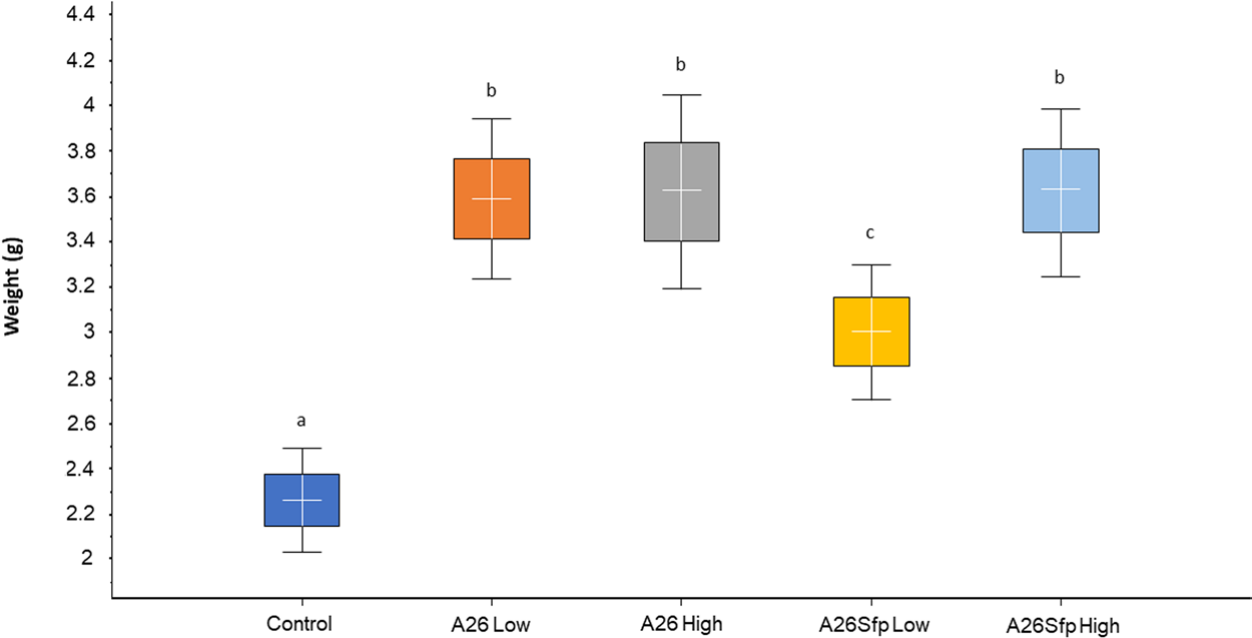
100 kernel weight (100-kw) in greenhouse assay. 100- kw after *Paenibacillus polymyxa* A26 and *P. polymyxa* A26∆sfp treatment at two inoculum densities, 10^6^ (Low) and 10^8^ (High) cells/ml. ANOVA univariate analysis was performed and post-hoc LSD tests used to control (p < 0.05). Different letters indicate statistically significant differences. Error bars represent standard deviations.

In order to confirm the results with A26∆sfp in reference to its wild-type A26 antagonistic abilities in the wheat grain assay, the experiment was performed in more natural settings, in the greenhouse. The winter wheat Stava seeds were vernalized and grown by standard procedures until the flowering stage. Then the wheat heads were sprayed with A26∆sfp and A26 at the two inoculum densities used in the wheat grain assay (10^6^ and 10^8^). Anthesis is the stage of the greatest susceptibility to FHB, as anthers are the common entry route into the plant. Hence, one week after the antagonist treatment (end of flowering stage) 30 µl of macroconidia suspension was used to inoculate a single central floret on each wheat head (four replicates each containing twenty plants). Wheat heads were harvested 21 days after the inoculation at the fully ripe stage and evaluated for disease incidence, disease severity and 100-kernel weight. A26 efficiently antagonises *F. graminearum* and the effect is not dependent on initial inoculum density (Figs 3–5 and Table S1). As shown by the disease incidence (DI), disease severity (DS) and 100- kernel weight (100-kw) (53, 10, and 3.5 respectively) the *F. graminearum* pathogen was equally well antagonised at both A26 inoculum densities (Figs 3–5 and Table S1). While highly similar antagonistic parameters of DI, DS and 100-kw (53, 10 and 3.6 respectively) were scored for A26∆sfp at the higher inoculum density (10^8^), up to 25% reduced antagonistic ability was observed at 10^6^ density (78, 30 and 2.9 respectively) (Figs 3–5 and Table S1). No disease symptoms were scored on plants after sole A26 treatment or after A26Sfp treatment, at either of the two inoculum densities, or after water treatment (Table S1). 100-kw of the efficient pathogen treatments was highly similar to the kernel weight of control treatments with sole A26 or with A26Sfp or water (3.4 ± 0.6 g).

### Wheat head biofilm assessment

**Figure 6:**
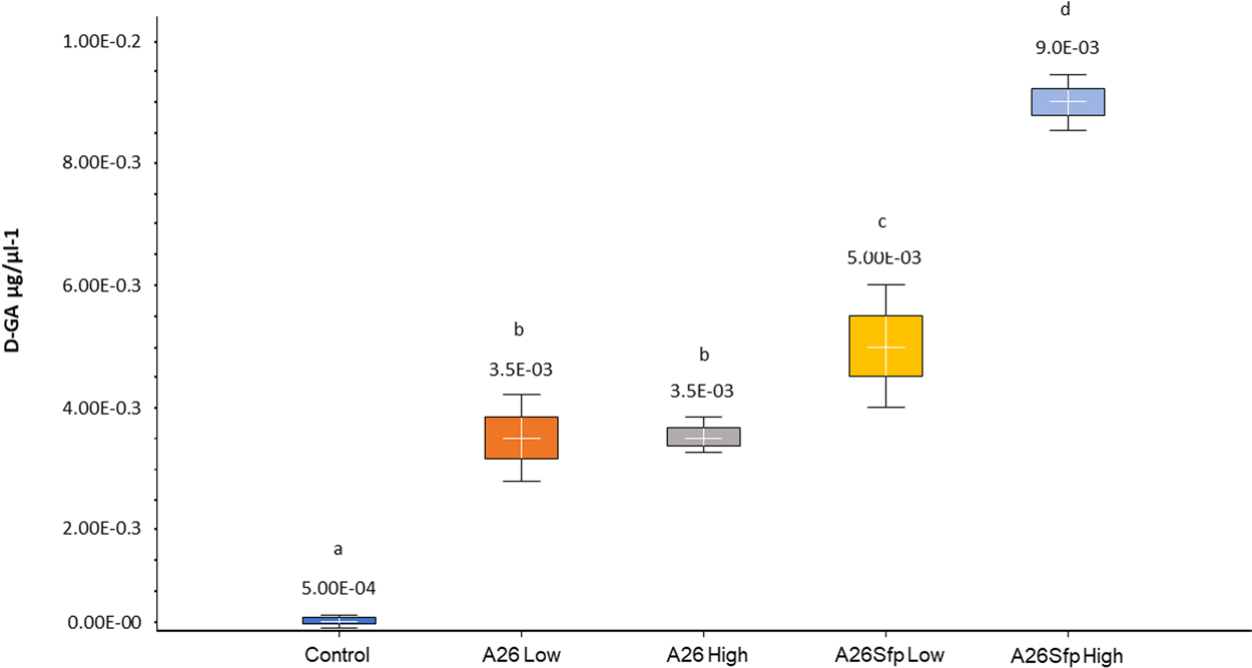
D- glucuronate (D-GA) content in wheat axis biofilms. D-GA content of *Paenibacillus polymyxa* A26 and A26∆sfp wheat axis biofilms at two inoculum densities, 10^6^ (Low) and 10^8^ (High) cells/ml. ANOVA univariate analysis was performed and post-hoc LSD test used to identify treatments significantly different from water control (p < 0.05). Different letters indicate statistically significant differences. Error bars represent standard deviations.

In order to follow the performance of A26∆sfp and its wild type A26, wheat heads were flushed with water immediately before the pathogen treatment in order to collect the biofilms developed after the A26 or A26∆sfp inoculations. The biofilm bacteria were re-isolated by selective plating, confirmed by PCR as described^13^, quantified, supernatant polysaccharides isolated and D-glucuronate content recorded (Table 2). A26∆sfp and its wild type A26 (10^3^ per ml) were re-isolated in the wheat head biofilms (Table 2). About 30% higher EPS content was detected in A26∆sfp biofilms in comparison to its wild type A26 strain at both initial inoculum densities (Table 2). While neither the EPS content of A26∆sfp nor that of A26 varied significantly according to the initial inoculum density, the D-GA content of the mutant was about 40% higher at 10^8^ inoculum density. The D-GA comprised 3.5% of its EPS at 10^6^ inoculum density while the content was 6% at 10^8^ inoculum density (Table 2 and Fig. 6). The D-GA content in A26 was significantly lower than in A26∆sfp. i.e. 3% of A26 EPS (Table 2 and Fig. 6). The D-GA content correlated with A26∆sfp antagonistic activity (r > 0.78 p < 0.01). Additionally, the EPS titre and its D-GA content in the native biofilm layers of the control plant spikes were evaluated (Fig. 6 and Table 2). The native biofilms of all the control spikes comprised around 6% D-GA (Table 2 and Fig. 6).

**Table 2:** EPS assessment in relation to bacterial and fungal growth.

	Bacterial population log CFU/ml^1^	EPS titre (µg/ml)	EPS D-GA content (%)	D-GA (µg/ml)	*F. graminearum* DNA titre (ng/µl)^2^
Control	1.19 ± 0.11^a1*^	0.8 ± 0.16^a^	6	0.05 ± 0.01^a^	13 ± 2.6
**Wheat spike wash**					
Wheat spike wash A26 (Low)^3^	3.12 ± 0.12^b^	10 ± 2^b^	3	0.3 ± 0.06^b^	<0.3
A26 (High)	3.09 ± 0.13^b^	10 ± 1.5^b^	3	0.3 ± 0.03^b^	<0.3
Wheat spike wash A26Sfp (Low)	3.02 ± 0.10^b^	14 ± 2.6^c^	3.5	0.5 ± 0.1^c^	4.5 ± 0.8
A26Sfp (High)	3.02 ± 0.09^b^	15 ± 2.4^c^	6	0.9 ± 0.05^d^	<0.3
**½ TSB cultures**					
½ TSB cultures A26	8.89 ± 0.16	10.05 ± 2.23	3	0.3 ± 0.05	ND
A26Sfp	8.69 ± 0.11	15.02 ± 2.57	6	0.9 ± 0.09	ND
Sodium alginate		15	82	8.3 ± 0.83	ND
Levan		15	0	ND	ND

^1^The bacteria were re-isolated by selective plating and confirmed by PCR^13^. ^1*^Counting of bacterial colony forming units was performed on ½ TSB plates

^2^*F. graminearum* DNA was isolated amplified and quantified using the Invitrogen™ Qubit™ 4 Fluorometer as described in Material and Methods.

^3^Two inoculum densities of A26 and A26Sfp (Low) −10^6^ and (High) 10^8^ cells per ml. Different letters indicate statistically significant differences (p < 0.05).

ND- not detected.

### *P. polyFig.myxa* A26∆sfp and *P. polymyxa* A26 polysaccharide fragment assessment

**Figure 7:**
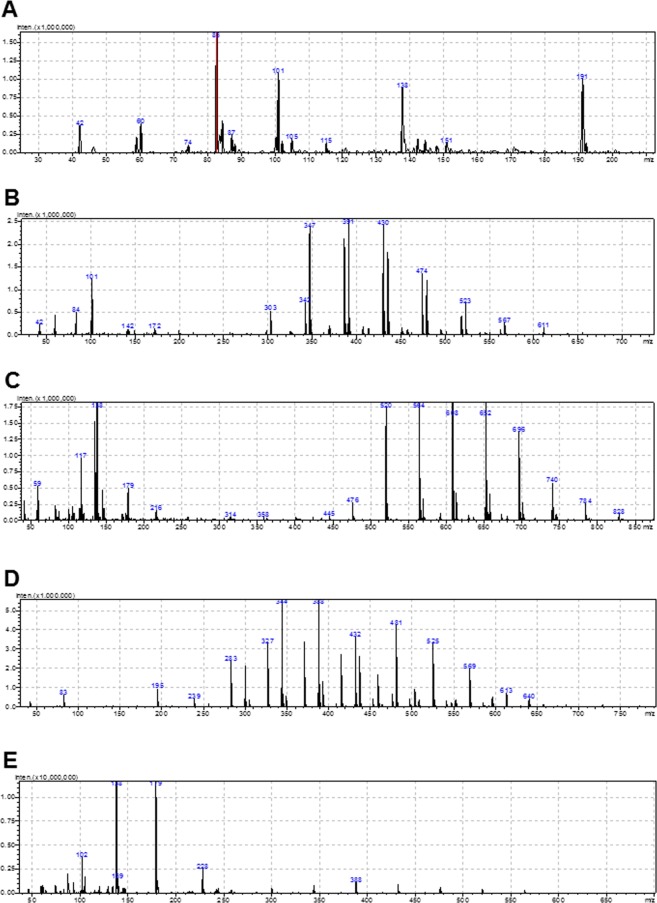
Typical examples of mass spectra recorded for diverse fractions of *Paenibacillus polymyxa* A26 and A26∆sfp wheat spike biofilms at low and high inoculum densities. Note the data presented also in Table 3 (A-I, B-II, C-III, D-IV, E-V). Aqueous solutions of biofilms (10 μl) were injected into the UHPLC–ESI-MS equipment. See Material and Methods.

UHPLC-ESI-MS under positive ionization was used to determine fragment ions. Figure 7 and Table 3 depict the Q1 mass spectra of the various major chromatographic fractions (Supplementary Information). In positive ion mode, no protonated molecules [M + H]^+^ were detected, but we clearly identified ammonium-adducted molecules [M + NH_4_]^+^, including those at m/z ratios of 191, 388, 567, and 722. The analyses demonstrated that A26 and A26Sfp biofilm EPS at low and high inoculum densities comprised monomers (176 Da), dimers (352 Da), trimers (528 Da) and tetramers (704 Da). There were no significant quantities of oligomers larger than tetramers.

**Table 3:** Fragment ions of the *P. polymyxa* A26 and *P. polymyxa* A26Sfp biofilm spectra.

Fraction/RT (min)	Oligomer	Fragment ions
I/2.9	monomer	191; 138; 101; 83; 42
II/3.3	trimer	567; 523; 474; 430; 491; 347; 303; 101; 84; 42
III/3.48	tetramer	784; 696; 652; 608; 564; 520; 476; 179; 138; 117
IV/3.68	dimer	388; 344; 283; 195; 83
V/4.15	tetramer	646; 562; 519; 475; 432; 388; 345; 302; 228; 179; 138; 87; 42

### Antagonistic activity of *P. polymyxa* A26∆sfp and A26 polysaccharides in the kernel assay

The greenhouse A26∆sfp treatment at the high inoculum density resulted in significant increases in spike biofilm EPS D-GA. This correlated with the treatment antagonistic activity. Hence, further studies were performed to study the effect using EPS isolated from A26∆sfp and A26 flask cultures, as well as commercial sodium alginate and levan as reference substrates with contrasting uronate content (Table 2). The results show that 15 µg/ml EPS treatments of both A26∆sfp and A26 efficiently antagonise the *F. graminearum* pathogen (Fig. 2B and Table S1). While the levan treatment revealed fungal overgrowth comparable to that in the *F. graminearum* control treatment, no pathogen was visually detected in the assay with commercial sodium alginate. *F. graminearum* growth, randomly confirmed by PCR assays followed by fluorometric quantification, revealed up to 0.3 ng/µl quantities of the pathogen DNA in A26∆sfp and A26 EPS and alginate assay versus more than 10 ng/µl with the levan and *F. graminearum* control treatment (Table S1).

### *P. polymyxa* A26∆sfp and A26 polysaccharide water holding capacity (WHC)

Addition of 0.6% (vol) biopolymers significantly increased WHC of sand. The A26 and A26Sfp enhanced the WHC about twice. The result is comparable to the enhancement by commercial sodium alginate WHC (Table 4). Levan treatment did not improve the sand WHC (Table 4).

**Table 4:** Effect of biopolymer amendment of sand soil WHC^1^ (%).

	Control	Sodium Alginate	Levan	A26Sfp	A26
WHC	27.6 ± 2.0^a2^	60.33 ± 2.0^b^	26.6 ± 2.1^a^	58.21 ± 3.1^b^	55.30 ± 2.0^b^

^1^WHC- Water Holding Capacity of sand 0.6%. biopolymer mixture. See Material and Methods. ^2^Different letters indicate statistically significant differences (p < 0.05).

## Discussion

Here we re-evaluated the modes of antagonism of the *P. polymyxa* wild type A26 and its mutant A26∆sfp against *F. graminearum* (Figs 2–5). While A26 antagonizes effectively at both low (10^6^ cells/ml) and high (10^8^ cells/ml) densities of inocula used in the study, A26∆sfp *F. graminearum* antagonism at 10^8^ inoculum density leads to up to 25% improved antagonistic ability, reaching the ability of the wild type to antagonize *F. graminearum* (Figs 2–5). At the same time, 10^3^ bacteria of both bacterial strains at both inoculum densities were recovered from the wheat heads one week after inoculation (Table 2). It is commonly known that even though bacteria are crucial for biofilm formation, their measurement is insufficient to quantify biofilms. The EPS which form the major part of the microbial biofilm matrix^19^ were detected in the spike biofilm water solutions. The A26 biofilm contains 1 µg and A26 Sfp 1.5 µg of EPS per ml (Table 2). The significant increase in the mutant biofilm matrix production is in accordance with our former findings^9^. Owing to the huge complexity of EPS matrix components, their detailed quantification is a challenge. D-glucuronate (D-GA) has previously been suggested as a proxy for biofilm comprehensive screening and uronic acid is widely determined as representative of myco-polysaccharides in biofilms^15^. The assay is based on the effectiveness of chemical bond disruption and digestion of polysaccharides into component monosaccharides^15^. Significant differences were detected in A26∆sfp D-GA at the two inoculum densities (Fig. 6 and Table 2). The D-GA comprised 6% of EPS at the 10^8^ inoculum density in comparison to 3.5% at the 10^6^ inoculum density (Fig. 6 and Table 2). The result correlates well with the ability of the mutant to antagonize *F. graminearum* (r > 0.78, p < 0.001). The EPS titre and its D-GA content in the native biofilm layers of the control plant spikes and of the spikes prior to A26 or A26Sfp treatment were evaluated (Fig. 6 and Table 2). It is interesting to note that all the native biofilms on control spikes, similar to A26Sfp high inocula biofilms, comprised around 6% D-GA. In order to further investigate the role of uronates, two commercially available reference polymers levan and alginic acid were used. Both EPS components can be found in *P. polymyxa* biofilms^20^ The commercial sugars contrast in D-GA content (Table 2). Levan is a polymer of fructose while alginate is comprised of uronates. While the alginate treatment fully antagonized the pathogen, fungal overgrowth was observed in the case of the levan treatment. The results further confirm the role of uronates regarding the antagonistic effect against *F. graminearum*.

How do we reconcile the outcome that 100 times initial inoculum density differences result in significant increases in the D-GA production of A26∆sfp? The interesting phenomenon that differences in initial inoculum density can lead to variable metabolism that cannot be linked to the inocula growth stage, has been reported earlier by Jeon *et al*.^21^. The *P. polymyxa* GBR-1 *β*-amylase gene was expressed only at a high inoculum density (10^8^ per ml). The gene was not expressed with the low density inoculum (10^6^)^21^. This phenomenon is certainly connected with complex biofilm biology and requires further study using parallel sequencing and high-resolution microscopy. However, even though the D-GA composition of the EPS matrix varies between the A26 and A26∆sfp strains, their isolated EPS, applied in surplus, induce resistance to *F. graminearum* in the wheat kernel assay (Fig. 2B).

It is widely recognised that uronic acid moieties affect the physiological and biological properties of polysaccharides. The uronates with high content of EPS, e.g. alginate and xanthan, result in increased water holding capacity^20^ It is believed that uronic acid backbones lead to changes in other sugar backbones, which eventually will result in alteration of their properties and bioactivity^22^. It is well known that EPS biosynthesis generally involves a very sophisticated network dependent on complex factors^23^. EPS are combinations of monomers and polymers consisting of sugar compounds connected via glyosidic linkages^14,24,25^. EPS length, composition and formation vary considerably^26–28^. The main factors that influence EPS interactions include the charges, polymer functional groups, backbone and chain fitness, and the relative concentrations of the constituents^14,24,25^. Hence EPS are not random co-polymers but vary and occur in combinations dependent on the various C and N sources used for bacterial growth^24,25^. We were interested in the fragment length of the EPS in A26 and A26∆sfp wheat head biofilms. The analyses demonstrated that in biofilms from both the strains, the primary compounds were monomers (176 Da), dimers (352 Da), trimers (528 Da) and tetramers (704 Da). There were no significant quantities of oligomers larger than tetramers (Fig. 7, Table 3).

Microorganisms in natural environments are subjected to various fluctuations in environmental conditions. It has been suggested that the ecological ‘success’ of EPS depends on their potential to favorably influence the microorganisms’ adaptation to the environment^29^. The EPS matrix serves as the microbial interface with the environment. In addition to environmental adaptation, EPS are also used for social skills communication, compartmentalization, competence and defense^19^. As the production of EPS requires copious amounts of energy, their regulatory control is important and there are many levels of EPS synthesis. Different subpopulations of *eps*- producing genes are activated during the different stages of biofilm development^30^. Several EPS molecules have high WHC which can be even as high as 15 times their weight^19,20^. WHC water accumulation can protect against water stress and was suggested by us as a mechanism of the drought tolerance enhancement of EC, SFS rhizobacterial strains^9,10^. The strains from the *Hordeum spontaneum* rhizosphere are likely to have machinery contributing to the wild cereal progenitors’ adaptation to the unfavorable environment^9^. In addition to the alleviation of plant drought stress, the strain WHC contributes to nutrient availability, maintaining water potential and physiological processes^19^. In our experiment the D-GA-containing EPS layer with high WHC could provide a physical barrier against *F. graminearum* pathogens. UHPLC-ESI-MS fragment assessments of the wheat head biofilm EPS after inoculation with A26 or A26Sfp at low and high inoculum densities showed that all biofilm EPS contain the mono-, di-, tri-, and tetramer fragment mixture (Fig. 7 and Table 3). Yet it is hard to speculate which types of D-GA-containing EPS were produced by the bacterial strains in the assay. It is clear that both A26∆sfp and A26 EPS are capable of absorbing water. The study in which sand was mixed with the biopolymers showed that A26∆sfp and A26 EPS have high WHC, which is similar to sodium alginate WHC (Table 4). It is interesting to note that the treatment with the commercial low titre (15 µg/ml^1^) reference substrate alginate resulted in protection against the pathogen in the model system. The result supports the idea that a biocontrol agent EPS matrix with high WHC is one of the critical factors providing a mechanism against *F. graminearum* pathogens. Several research groups have considered the strategy of application of pure EPS^19^. The work has mostly not resulted in consistent results owing to the complexity of the physical and chemical conditions of the natural environments as well as to the presence of native microbial communities which metabolize the EPS^19^. On the other hand, it is possible to engineer the EPS production of the native biofilm formers via the nutrient supply^14^.

Here we present a study where the initial observations of the antagonism of the *P. polymyxa* A26 biofilm to *F. graminearum* in the kernel assay are examined at a coarser scale in the greenhouse, and at a finer scale in experiments with commercial EPS application. We show that A26 biofilm EPS are capable of antagonizing *F. graminearum* and that the uronate content of the EPS is of critical importance in the antagonism.

It is clear that fundamental understanding of the genes and mechanisms of real-time biofilm formation is needed to explain the performance of the *P. polymyxa* native isolate A26 and its mutant A26∆sfp. Previous studies have shown that the multifaceted information about the bacterial biofilm cannot simply be adopted from studies of their domesticated strains^31^. It is possible that the A26 uronate-containing EPS matrix is required for efficient antagonism in the cases where the more common mechanisms of antagonism with antibiotic lipopeptides^4,9^ are not available (as with A26∆sfp). This would be in accordance with the fact that surplus EPS was used in the grain assay. How much the EPS produced by wild type *P. polymyxa* A26 contribute to its *F. graminearum* antagonism under different environmental conditions remains to be elucidated. The study here indicates that along with the lipopeptides^4,9^, the bacterial biofilm EPS compounds are capable of antagonizing *F. graminearum* and that the uronate content of the polysaccharides is of critical importance.

Bacterial EPS is a promising class of sustainable biopolymers to meet various industrial/agricultural requirements. Considering the presence of bacterial natural surface biofilms and that only about 1% of the bacteria can at present be cultured, many EPS are yet to be identified^23,32^. Even though the EPS layer is dependent upon the perception of numerous environmental signals from the host and the ecosystem, this would open the new range of application of EPS in integrated pathogen management protecting against stress factors under climate change.

## Conclusions

*Paenibacillus polymyxa* A26 represents a promising biocontrol agent for addressing the many challenges *F*. *graminearum* poses to agricultural crop yield and quality. However, to capitalize on this potential, an improved understanding of the mechanism of action is needed. This study demonstrates that the biofilm polysaccharides containing D-GA significantly contribute to antagonism and that the initial inoculum density plays a significant role in this.

## Supplementary information

Supplementary Info
