# Supplementary material for: Paenibacillus polymyxa biofilm polysaccharides antagonise Fusarium graminearum
Source: Sci Rep. 2019 Jan 24;9:662. doi: 10.1038/s41598-018-37718-w (PMC6345971; doi:10.1038/s41598-018-37718-w)
Supplement: Supplementary file 1 — Supplementary Info [file 41598_2018_37718_MOESM1_ESM.docx]

***Paenibacillus polymyxa*  biofilm polysaccharides antagonise *Fusarium graminearum***

Salme Timmusk^1*^, Dana Copolovici^2^, Lucian Copolovici^2^, Tiiu Teder^1,3^ Eviatar Nevo^4,5^, and Lawrence Behers^1,6^

^1^Department of Forest Mycology and Plant Pathology, Swedish University of Agricultural Sciences, Uppsala, Sweden

^2^Faculty of Food Engineering, Arad University of Aurel Vlaicu, Arad, Romania

^3^Department of Plant Physiology, Estonian University of Life Sciences, Tartu, Estonia

^4^International Graduate Centre of Evolution, University of Haifa, Israel,

^5^National Academy of Sciences, USA

**^6^**Nova West Technologies & Communications, Tucson, AZ, USA

**Supplementary Information**

**I. Table S1 *Fusarium graminearum* quantification assay**^1^

| **Experimental series** |  | ***F. graminearum***  **CFU** | **Antagonist CFU/EPS titre (µg/ml)** | ***F. graminearum* DNA titre (ng/µl)** |
| --- | --- | --- | --- | --- |
|  |  |  | |  |
| 1 | Kernel assay | 10^3^ | 0 | 12±2.6 |
|  |  | 10^3^ | A26Sfp 10^6^ | 3.5±0.6 |
|  |  | 10^3^ | A26Sfp 10^8^ | <0.3 |
| 2 | Wheat head assay | 10^3^ | 0 | 13±2.6 |
|  |  | 10^3^ | A26 10^6^ | <0.3 |
|  |  | 10^3^ | A26 10^8^ | <0.3 |
|  |  | 10^3^ | A26Sfp 10^6^ | 4.5±0.8 |
|  |  | 10^3^ | A26Sfp 10^8^ | <0.3 |
|  |  | 0 | A26 10^6^ | ND |
|  |  | 0 | A26 10^8^ | ND |
|  |  | 0 | A26Sfp 10^6^ | ND |
|  |  | 0 | A26Sfp 10^8^ | ND |
|  |  | 0 | Sterile water | ND |
| 3 | Kernel EPS assay | 10^3^ | 0 | 13±2.9 |
|  |  | 10^3^ | A26 15 | <0.3 |
|  |  | 10^3^ | A26Sfp 15 | <0.3 |
|  |  | 10^3^ | Levan 15 | 11±2.0 |
|  |  | 10^3^ | Sodium alginate 15 | <0.3 |
|  |  |  |  |  |

^1^ *F. graminearum* DNA was isolated amplified and quantified using the Invitrogen™ Qubit™ 4 Fluorometer as described in Material and Methods

CFU colony forming units of inoculants. Spores were used for *F. graminearum* and vegetative cells for antagonists inoculation

EPS extracellular polysaccharides; ND not detected

**II.Major chromatographic fractions *P. polymyxa* A26 and *P. polymyxa* A26Sfp biofilms**

**Qualitative determination of biofilm composition was performed by UHPLC-MS as described in Material and Methods**

The chromatographic analyses have been performed using a liquid chromatograph (Nexera X2, Shimadzu, Tokyo, Japan) equipped with a diode array detector (M30A, Shimadzu, Tokyo, Japan) and a mass spectrometer (Model 8040, Shimadzu, Tokyo, Japan). The separation of compounds was performed on Nucleodur 100-5-NH2-RP columns (4.6 mm i.d. x 250 mm column length, 5 µm particle size, Macherey-Nagel GmbH, Duren, Germany). The column temperature was maintained at 35 ºC and the flow rate at 1 ml min^-1^. The solvents used for the chromatographic elution consisted of ultra-pure water with 0.1% TFA (A) and acetonitrile (B). The chromatographic elution program used was an isocratic one, with 25% A and 75% B, for 25 minutes. The injected volume of sample and standards was 10 µl. The DAD detector spectra were recorded between 200 and 600 nm. The mass spectrometer was equipped with an electrospray ionization (ESI) source operated in positive ion mode, and quantification was carried out in multiple reaction monitoring (MRM) mode. The mass range was between m/z 15 and 1990. The ion spray temperature was maintained at 250 °C. The drying gas flow rate was 10 L/min.

S45 0 1

Peak# Ret. Time Area Height Mark Conc. Unit ID# Name Area%

1 1.791 32871 7291 M 4.589 4.589

2 1.933 42783 9838 V M 5.973 5.973

3 2.802 10881 2465 M 1.519 1.519

4 2.882 18864 4608 V M 2.634 2.634

5 2.945 12461 3163 V M 1.740 1.740

6 3.087 125973 42489 M 17.587 17.587

7 3.139 444767 54744 V M 62.094 62.094

8 3.466 11110 2296 M 1.551 1.551

9 3.589 1034 45 M 0.144 0.144

10 4.183 15537 1613 M 2.169 2.169

Total 716280 128552 100.000 100.000

RT 1.79m

RT 1.93m

RT 2.7 m

RT 2.85m

RT 2.93m

RT 3.3m

RT 3.48m

RT 3.68m

RT 3.18m

RT 4.12m

RT 4.17m

RT 5.21m

RT 6.56m

RT 8.8 m

RT 9.6m

RT 11.76m

RT14.05m

RT 14.28m

RT 18.08m

RT 19.10m

RT 22.08m

S45 0 2

Peak# Ret. Time Area Height Mark Conc. Unit ID# Name Area%

1 1.788 34033 7511 M 4.770 4.770

2 1.931 41198 10985 V M 5.774 5.774

3 2.797 15910 2696 M 2.230 2.230

4 2.878 20892 4955 V M 2.928 2.928

5 2.946 17941 4910 V M 2.515 2.515

6 3.087 192662 51665 M 27.004 27.004

7 3.145 361832 48890 V M 50.716 50.716

8 3.458 10984 2354 M 1.540 1.540

9 3.589 659 43 M 0.092 0.092

10 4.190 16321 1693 M 2.288 2.288

11 24.896 1016 135 0.142 0.142

Total 713448 135837 100.000 100.000

S45 1

Peak# Ret. Time Area Height Mark Conc. Unit ID# Name Area%

1 1.793 33976 8002 M 4.783 4.783

2 1.934 36145 9830 V M 5.088 5.088

3 2.802 12566 2342 M 1.769 1.769

4 2.882 20277 5047 V M 2.854 2.854

5 2.948 14634 3705 V M 2.060 2.060

6 3.099 125921 43549 M 17.725 17.725

7 3.141 443238 54245 V M 62.393 62.393

8 3.468 9444 1950 M 1.329 1.329

9 3.568 220 -42 M 0.031 0.031

10 4.190 13981 1452 M 1.968 1.968

Total 710401 130080 100.000 100.000

S 45 2

Peak# Ret. Time Area Height Mark Conc. Unit ID# Name Area%

1 1.794 29492 7377 M 4.198 4.198

2 1.935 32652 9430 V M 4.647 4.647

3 2.802 10938 2179 M 1.557 1.557

4 2.883 18899 4845 V M 2.690 2.690

5 2.951 14079 3585 V M 2.004 2.004

6 3.092 124667 43336 M 17.744 17.744

7 3.148 446123 53667 V M 63.497 63.497

8 3.466 8387 1852 M 1.194 1.194

9 3.589 410 51 M 0.058 0.058

10 4.199 16945 1683 M 2.412 2.412

Total 702590 128005 100.000 100.000

S 49 01

Peak# Ret. Time Area Height Mark Conc. Unit ID# Name Area%

1 1.797 21156 5159 M 2.928 2.928

2 1.938 55541 14091 V M 7.686 7.686

3 2.117 403 269 M 0.056 0.056

4 2.254 4798 1776 M 0.664 0.664

5 2.804 10028 2262 M 1.388 1.388

6 2.882 17662 4856 V M 2.444 2.444

7 2.952 16708 3874 V M 2.312 2.312

8 3.139 421717 59638 M 58.361 58.361

9 3.216 141755 39566 V M 19.617 19.617

10 3.467 13646 2962 M 1.888 1.888

11 3.600 939 59 M 0.130 0.130

12 4.162 18244 1675 M 2.525 2.525

Total 722598 136187 100.000 100.000

RT 2.24m

S 49 0 2

Peak# Ret. Time Area Height Mark Conc. Unit ID# Name Area%

1 1.796 19758 5010 M 2.721 2.721

2 1.938 50909 13718 V M 7.011 7.011

3 2.254 4282 1705 M 0.590 0.590

4 2.800 14264 2719 M 1.964 1.964

5 2.884 19395 4666 V M 2.671 2.671

6 2.950 10950 3286 V M 1.508 1.508

7 3.088 71380 33694 M 9.830 9.830

8 3.134 506672 57054 V M 69.775 69.775

9 3.465 15178 2949 M 2.090 2.090

10 3.600 600 -25 M 0.083 0.083

11 4.170 12763 1493 M 1.758 1.758

Total 726153 126269 100.000 100.000

RT 2.3 m

S49 1

Peak# Ret. Time Area Height Mark Conc. Unit ID# Name Area%

1 1.800 20243 5337 M 2.879 2.879

2 1.941 53279 14086 V M 7.578 7.578

3 2.118 243 167 M 0.035 0.035

4 2.257 4760 1651 M 0.677 0.677

5 2.803 6551 1308 M 0.932 0.932

6 2.885 14346 3738 V M 2.041 2.041

7 2.955 8321 2543 V M 1.184 1.184

8 3.140 331309 58333 M 47.124 47.124

9 3.184 239664 52398 V M 34.089 34.089

10 3.474 13213 2674 M 1.879 1.879

11 3.635 854 231 M 0.121 0.121

12 4.179 10272 1284 M 1.461 1.461

Total 703056 143751 100.000 100.000

S 49 0 2 sec

Peak# Ret. Time Area Height Mark Conc. Unit ID# Name Area%

1 1.796 20826 5131 M 2.873 2.873

2 1.938 54361 13992 V M 7.500 7.500

3 2.254 4598 1746 M 0.634 0.634

4 2.800 12821 2617 M 1.769 1.769

5 2.884 17157 4637 V M 2.367 2.367

6 2.950 13370 3316 V M 1.845 1.845

7 3.134 572983 56665 M 79.055 79.055

8 3.465 14967 2937 M 2.065 2.065

9 3.611 644 6 M 0.089 0.089

10 4.170 13061 1507 M 1.802 1.802

Total 724788 92555 100.000 100.000

S 49 2 at 190 nm

Peak# Ret. Time Area Height Mark Conc. Unit ID# Name Area%

1 1.801 19523 5238 M 2.801 2.801

2 1.941 53561 14119 V M 7.683 7.683

3 2.257 4134 1590 M 0.593 0.593

4 2.805 6424 1284 M 0.922 0.922

5 2.886 16571 3975 V M 2.377 2.377

6 2.956 7022 2410 V M 1.007 1.007

7 3.140 568440 61766 M 81.541 81.541

8 3.473 11188 2434 M 1.605 1.605

9 3.600 882 -23 M 0.127 0.127

10 4.170 9375 1137 M 1.345 1.345

Total 697120 93929 100.000 100.000

Peak# Ret. Time Area Height Mark Conc. Unit ID# Name Area%

1 1.801 19523 5238 M 2.801 2.801

2 1.941 53561 14119 V M 7.683 7.683

3 2.257 4134 1590 M 0.593 0.593

4 2.805 6424 1284 M 0.922 0.922

5 2.886 16571 3975 V M 2.377 2.377

6 2.956 7022 2410 V M 1.007 1.007

7 3.099 84149 40956 M 12.071 12.071

8 3.140 284255 61766 V M 40.776 40.776

9 3.195 200036 47392 V M 28.695 28.695

10 3.473 11188 2434 M 1.605 1.605

11 3.600 882 -23 M 0.127 0.127

12 4.170 9375 1137 M 1.345 1.345

Total 697120 182276 100.000 100.000

RT 2.93m

RT 3.3 m

RT 3.48 m

RT 3.7 m

RT 4.55m

RT 5.07m

RT 6.85m

RT 7.5 m

RT 8.9 m

RT 9.56 m

RT 9.9 m

RT 12.45m

RT 13.5m

RT 15.45m

RT 17.8m

RT 17.97m

RT 24.7m

S45 0 1

RT 1.65m
